# MRI-guided adaptive stereotactic body radiotherapy for the prostate bed: a prospective study of safety, tolerability, and patient-reported quality-of-life outcomes

**DOI:** 10.1016/j.ctro.2026.101241

**Published:** 2026-07-25

**Authors:** Daniela Gonsalves Pieretti, Fernando López-Campos, Abrahams Ocanto, Maria González, Lisselott Torres, Castalia Fernandez, Gloria Sanchez, Gloria Guardia, Alvaro Flores, Jon Andreescu, Laura Viduera, Dulce Urias, Ofelia Cabanes, Julia Rogriguez, Sigfredo Elias Romero Zoghbi, Jose Antonio González, Miren Gaztañaga, Luis Glaria, Maia Dzhugashvili, Stefano Arcangeli, Federica Ferrario, Juan Ignacio Martinez Salamanca, Giulio Francolini, Felipe Couñago

**Affiliations:** a Department of Radiation Oncology, GenesisCare-Vithas La Milagrosa University Hospital, 28010 Madrid, Spain; b Department of Radiation Oncology, GenesisCare-San Francisco de Asís University Hospital, 28002 Madrid, Spain; c Department of Medicine, School of Medicine, Health and Sport, European University of Madrid, 28670 Madrid, Spain; d Department of Radiation Oncology, Ramón y Cajal University Hospital, 28034 Madrid, Spain; e Department of Radiation Oncology, GenesisCare Campo Gibraltar, 11370 Algeciras, Spain; f Department of Radiation Oncology, GenesisCare Hospital San Juan de Dios, 14012 Cordoba, Spain; g Department of Radiophysics, GenesisCare-Vithas La Milagrosa University Hospital, 28010 Madrid, Spain; h Department of Radiophysics, GenesisCare-San Francisco de Asís University Hospital, 28002 Madrid, Spain; i Department of Radiation Oncology, Instituto Nacional de Cancerología, 14080 Ciudad de Mexico, Mexico; j Department of Radiation Oncology, GenesisCare Talavera de la Reina, 45600 Talavera de la Reina, Spain; k Department of Radiation Oncology, GenesisCare Sevilla, 41092 Sevilla, Spain; l Department of Radiation Oncology, Hospital Universitario La Paz, 28046 Madrid, Spain; m School of Medicine and Surgery, University of Milan Bicocca, Milan, Italy; n Radiation Oncology, Fondazione IRCCS San Gerardo dei Tintori, Monza, Italy; o Department of Urology, Lyx Institute of Urology, Universidad Francisco de Vitoria, 28223 Madrid, Spain; p Radiation Oncology Unit, Oncology Department, Careggi University Hospital, University of Florence, Florence, Italy; q Radiation Oncology Department, Hospital Clínico San Carlos, 28040 Madrid, Spain

**Keywords:** Prostate cancer, Stereotactic body radiotherapy, MR-guided radiotherapy, Prostate bed, Biochemical recurrence, Patient-reported outcomes, Quality of life, Adaptive radiotherapy, MR-LINAC

## Abstract

**Background and objectives:**

Magnetic resonance-guided radiotherapy (MRgRT) applied as stereotactic body radiotherapy to the prostate bed represents an emerging post-prostatectomy treatment approach, offering superior soft-tissue visualization and the potential for daily online adaptive planning. This study reports a prospective experience with MR-guided adaptive SBRT to the prostate bed, with a primary focus on treatment-related adverse events and patient-reported quality-of-life (QoL) outcomes.

**Materials and methods:**

A prospective cohort of 31 patients with biochemical recurrence following radical prostatectomy was treated with MR-guided adaptive SBRT (32.5 Gy in 5 fractions) using a 0.35 T MR-LINAC (MRIdian®, ViewRay). Elective pelvic nodal irradiation was delivered in 42% of patients (25 Gy in 5 fractions), and a simultaneous integrated boost (SIB) to macroscopic disease (35 Gy in 5 fractions) was administered in 9.7% of cases. Concomitant androgen deprivation therapy (ADT) was administered in 77.4% of patients. Adverse events were graded according to the Common Terminology Criteria for Adverse Events (CTCAE) version 5.0, and patient-reported outcomes were assessed using the Expanded Prostate Cancer Index Composite-26 (EPIC-26) and the International Prostate Symptom Score (IPSS).

**Results:**

With a median follow-up of 8.8 months (IQR: 6.1–16.8), no grade ≥ 3 adverse events were observed at any time point. Acute grade ≥ 2 genitourinary (GU) adverse events were **3.3%** at treatment completion (1/30) and 4.3% at 6 months (1/23), with low-grade urinary incontinence as the predominant event. “No grade ≥ 2 gastrointestinal (GI) adverse events were observed throughout the follow-up period.EPIC-26 urinary domain scores were preserved over time (baseline: 79.6; 6 months: 81.5), while bowel domain scores remained consistently at ceiling levels (median 100 points at all time points). Median prostate-specific antigen (PSA) declined from 0.31 ng/mL pre-treatment to 0.02 ng/mL at 6 months, with continued suppression at 12 months (0.04 ng/mL, *n* = 11). Biochemical response was 100% (31/31), although interpretation is limited by the short follow-up.

**Conclusions:**

MRgRT to the prostate bed demonstrates a favorable early safety and tolerability profile, with no grade ≥ 3 adverse events, low rates of grade ≥ 2 GU adverse events (4.3% at 6 months, 1/23), and absence of late grade ≥ 3 GI adverse events, while maintaining patient-reported QoL outcomes.

## Highlights

• MR-guided adaptive SBRT to the prostate bed is feasible and well tolerated.
• No grade ≥ 3 adverse events were observed at any time point.
• Grade ≥ 2 GU adverse events: 3.3% at end-of-treatment, 4.3% at 6 months.
• EPIC-26 urinary and bowel domain scores were preserved throughout follow-up.
• Prospective PRO data support further investigation of MRgRT in this setting.

## 1 Introduction

Stereotactic body radiotherapy (SBRT) has become an established treatment paradigm in localized prostate cancer, supported by the putatively low α/β ratio of prostate adenocarcinoma and significant advances in image-guided radiotherapy (IGRT). Randomized trials and large clinical series have demonstrated that SBRT provides excellent biochemical control with low rates of adverse events, while substantially reducing overall treatment duration, thereby improving treatment efficiency and patient convenience [1], [2], [3], [4], [5], [6].

Emerging retrospective and early prospective series have suggested feasibility of post-prostatectomy SBRT, although with heterogeneous adverse event outcomes [7], [8], [9]. The emergence of magnetic resonance imaging-guided radiotherapy (MRgRT) has further advanced treatment delivery by enabling superior soft-tissue contrast, intrafraction motion visualization, and online adaptive planning [10]. This technology offers distinct advantages over conventional computed tomography (CT)-guided radiotherapy, including improved target and organs of interest (OAR) delineation and the potential for planning target volume (PTV) margin reduction [11]. The MIRAGE phase III trial demonstrated that MRgRT significantly reduced acute grade ≥ 2 genitourinary (GU) adverse events compared with CT-SBRT (24.4% vs. 43.4%, *p* = 0.01) in patients with intact prostate [12], providing a strong clinical and dosimetric rationale for exploring MRI guidance in the post-prostatectomy setting, although direct extrapolation should be made with caution given anatomical and biological differences.

Postoperative radiotherapy remains a cornerstone in the management of prostate cancer following radical prostatectomy, particularly for patients with adverse pathological features or biochemical recurrence. While the RADICALS-RT trial established that moderately hypofractionated radiotherapy (52.5 Gy in 20 fractions) yields patient-reported outcomes (PROs) comparable to conventional fractionation (66 Gy in 33 fractions) [2], the role of SBRT in this specific anatomical context remains investigational and requires careful evaluation of both efficacy and long-term safety [13]. When delivering SBRT to the prostate bed, a critical challenge is managing intrafraction motion, which can compromise dose precision and increase exposure to surrounding OAR. MRgRT enables real-time cine imaging and gating strategies, allowing continuous monitoring and mitigation of target motion without the need for implanted fiducials [14], [15].

Despite increasing international experience with post-prostatectomy SBRT, prospective data on MR-LINAC platforms remain scarce, particularly regarding patient-reported outcomes [16]. To address the evidence gap in the primary setting, we conducted a prospective study evaluating the safety and tolerability of MRgRT to the prostate bed in patients with biochemical recurrence, with a predefined focus on adverse events and patient-reported quality-of-life (QoL) outcomes. The primary endpoint was acute and subacute adverse events, with secondary endpoints including patient-reported QoL and early biochemical response.

## 2 Materials and methods

### 2.1 Study design and patient population

This investigation represents a prospective cohort analysis derived from an ongoing basket-type protocol, conducted at Hospital La Milagrosa, which received ethical approval from the Ethics Committee of Hospital Universitario de La Princesa (registry #5289). The basket-type protocol encompasses patients receiving MRgRT using a 0.35 T MR-LINAC system (MRIdian®, ViewRay) for various indications; the present analysis is restricted to the prostate bed cohort. For the present analysis, patients treated between July 2023 and May 2024 were selected according to pre-specified inclusion criteria and a predefined analysis cut-off date.

Eligible participants were men aged ≥18 years with histologically confirmed prostate cancer following radical prostatectomy, with or without lymphadenectomy, presenting with biochemical recurrence (defined as two consecutive PSA values >0.20 ng/mL separated by ≥2 months and occurring ≥6 months after surgery), and ECOG performance status 0–1. Biochemical recurrence alone was considered a sufficient indication for prostate bed radiotherapy. The presence of macroscopic local recurrence or pelvic nodal disease on staging imaging was used to determine treatment volumes and boost strategies (see Section 2.2). All patients underwent staging imaging including pelvic MRI and, when available, PSMA-PET/CT. Macroscopic local recurrence was defined as a lesion identified within the prostate bed on pelvic MRI and/or PSMA-PET/CT. Pelvic nodal recurrence was defined as PSMA-positive pelvic lymph node(s) detected on PSMA-PET/CT. Biochemical recurrence alone was considered a sufficient indication for prostate bed radiotherapy, whereas the presence of macroscopic local or nodal disease determined the use of boost volumes and/or pelvic nodal irradiation. Exclusion criteria included documented distant metastatic disease and prior pelvic radiotherapy.

Concomitant androgen deprivation therapy (ADT) was prescribed according to the risk-stratification protocol described by González-San Segundo et al. [17]: low-risk patients received no ADT, intermediate-risk patients received 0–6 months, and high-risk patients received 6 months to 2 years. Elective pelvic lymph node irradiation (WPRT) was added for pN0 patients with high-risk features (Gleason score 8–10 and post-prostatectomy PSA nadir >0.4 ng/mL), in line with available evidence from retrospective series [18], [19] and randomized data such as the SPPORT trial [5].

### 2.2 MRI-guided radiotherapy treatment planning

Following written informed consent, patients underwent a standardized bowel and bladder preparation protocol, including at least one week of low-residue diet and laxatives. Simulation was performed using a TRUFI MRI sequence with controlled bladder filling and rectal emptying, supplemented by a planning CT for electron density mapping.

Target and OAR contouring followed RTOG consensus guidelines [20], [21] for the prostate bed and pelvic lymph nodes. Per RTOG guidelines, the posterior bladder wall was included within the prostate bed CTV, as the prostate bed is a virtual space physiologically occupied in part by the bladder. The prostate bed CTV was contoured with Boolean subtraction of the bladder lumen during both initial planning and daily adaptive workflows, while the nodal CTV similarly excluded bowel structures.

Planning Target Volumes (PTV)margins were 2 mm for the prostate bed or macroscopic disease (PTV1, PTV2) and 4 mm for pelvic lymph nodes (PTV2), reflecting the use of daily adaptive MR guidance. All patients received 32.5 Gy in 5 fractions to PTV1 (prostate bed). When elective nodal irradiation was indicated, 25 Gy in 5 fractions was delivered to the pelvic lymph node volume (PTV2). A simultaneous integrated boost (SIB) to 35 Gy in 5 fractions (PTV3) was delivered to any macroscopic disease identified on MRI or PSMA-PET/CT, including both prostate bed recurrences and PSMA-positive pelvic lymph nodes. The planning objective required that ≥95% of the PTV received the prescription dose, with hotspots limited to <110%. Per institutional protocol, coverage below this threshold (V95% < 95%) was accepted when required to fulfil organ-at-risk constraints during online adaptive replanning.

### 2.3 Adaptive delivery protocol

During adaptive delivery, all anatomical structures were reviewed and, when necessary, re-contoured daily according to predefined institutional protocols. Online replanning was performed when OAR constraints were not met.

A rectal surrogate structure, delineated with ≥1 cm cranio-caudal margins relative to the PTV, was used for gating and continuously monitored using cine MRI in sagittal and coronal planes (region-of-interest (ROI) threshold: 5–7%; confidence level: 85–95%).

All fractions were delivered using an online adaptive workflow. The median total time per fraction from patient entry to treatment completion, including set-up MRI acquisition, daily adaptive replanning, and beam delivery, was 30 min (range 20–75 min). Secondary quality assurance (QA) was performed at each fraction using ArcCHECK® (Sun Nuclear Corp.) dosimetric verification, ensuring treatment delivery accuracy.

### 2.4 Follow-up and outcome assessment

Physician-assessed adverse events were graded according to CTCAE version 5.0, and classified as acute (occurring ≤90 days from the start of radiotherapy) or late (>90 days from the start of radiotherapy). A clinically significant change in IPSS was defined as a change of ≥3 points from baseline, consistent with established thresholds in the literature. Serum PSA levels were obtained at baseline, 1–3 months post-treatment, every 3 months during the first year, and every 6 months thereafter.

PROs were collected using the International Prostate Symptom Score (IPSS) and the Expanded Prostate Cancer Index Composite-26 (EPIC-26) at baseline, 1–3 months post-treatment, and every 6 months thereafter for a planned minimum follow-up of 5 years.

### 2.5 Statistical analysis

Demographic, clinical, and treatment variables were prospectively recorded. Continuous variables are reported as medians with interquartile ranges (IQR), while categorical variables are presented as absolute and relative frequencies. Longitudinal changes in PRO were analyzed using linear mixed-effects models, accounting for repeated measures at baseline, 3, 6, and 12 months. Median follow-up was calculated from the date of first treatment fraction to the date of last follow-up using the reverse Kaplan–Meier method. Minimally important difference (MID) thresholds applied for EPIC-26 domains were: urinary function 6 points, urinary bother 5 points, bowel function 4 points, and sexual function 10 points, in accordance with published literature [22]. A clinically meaningful change in IPSS was defined as ≥3 points from baseline. All statistical analyses were performed using IBM SPSS Statistics version 26.0 (IBM Corp., Armonk, NY, USA), with a two-sided significance level set at *p* < 0.05.

## 3 Results

### 3.1 Patient and treatment characteristics

A total of 31 patients treated with MRgRT to the prostate bed were included. After a median follow-up of 8.8 months (IQR, 6.1–16.8), most patients had an ECOG performance status of 0 (83.9%). The cohort was mainly composed of intermediate-risk patients, with 38.7% classified as favorable and 16.1% as unfavorable, while 35.5% had high-risk disease.

Pathologically, Grade Group II was the most frequent finding, and tumors were predominantly organ-confined (67.7%), although extracapsular extension (pT3a), seminal vesicle invasion (pT3b), and nodal involvement (pN1) were each present in 16.1% of cases. Staging imaging was performed in all patients; PSMA-PET was evaluable in 29 patients, of whom 21 (72.4%) underwent the study (two patients were not evaluable due to renal insufficiency contraindicating radiotracer administration). Of the 31 treated patients: 15 (48.4%) had biochemical recurrence without macroscopic disease on staging imaging and received prostate bed irradiation alone; 13 (41.9%) received additional elective pelvic nodal irradiation (PTV2, 25 Gy) based on pN1 at surgery (*n* = 5) or imaging-detected nodal recurrence (*n* = 8); and 3 (9.7%) received a simultaneous integrated boost to macroscopic residual disease (PTV3, 35 Gy), of whom 2 had PSMA-positive nodal disease and 1 had visible prostate bed recurrence on MRI. Concomitant ADT was administered in 77.4% of patients. Detailed characteristics are summarized in Table 1.

**Table 1:** Patient, histopathological, and treatment planning characteristics of the study cohort.

Variable	Overall (*n* = 31)	Value
**Demographics**		
Age (y), median (range)	70	50–82
ECOG PS 0	26 (83.9%)	
ECOG PS 1	5 (16.1%)	
Median follow-up (mo), median (range)	8.8	1.7–21.5

**PSA**		
PSA at initial diagnosis (ng/mL), median (range)	4.97	0.13–68
Pre-RT PSA (ng/mL), median (range)	0.31	0.01–13.5

**Pathologic features**		
Pathologic T stage		
pT2 (all subtypes)	21 (67.8%)	
pT3a (ECE)	5 (16.1%)	
pT3b (SVI)	5 (16.1%)	

Pathologic N stage		
pN0	26 (83.9%)	
pN1	5 (16.1%)	

Pathologic Gleason		
I	4 (12.9%)	
II	11 (35.5%)	
III	7 (22.6%)	
IV	5 (16.1%)	
V	4 (12.9%)	

**Risk classification**		
Low risk	2 (6.5%)	
Favorable intermediate risk	12 (38.7%)	
Unfavorable intermediate risk	5 (16.1%)	
High risk	11 (35.5%)	
Isolated biochemical recurrence	1 (3.2%)	

**Advanced imaging**		
PSMA-PET performed (of 29 evaluable patients)	21 (72.4%)	

**SBRT parameters**		
Prostate bed dose (Gy), median (range)	32.5	32.5–32.5
Dose per fraction (Gy)	6.5	
Number of fractions	5	
PTV1 volume (cc), median (range)	63.6	38.5–127.7
PTV1 V95% coverage (%), median (range)	84.3	62.5–98.2
Prostate bed boost (SIB to PTV3, 35 Gy)	3 (9.7%)	
Elective pelvic nodal RT (PTV2, 25 Gy)	13 (41.9%)	
Overall treatment duration (d), median (range)	9	7–39

**Systemic therapy**		
Concurrent ADT use	24 (77.4%)	
Duration 6 months	9 (37.5%)	
Duration 18 months	2 (8.3%)	
Duration 24 months	13 (54.2%)	

ECOG PS, Eastern Cooperative Oncology Group performance status; RT, radiotherapy; IQR, interquartile range; ADT, androgen deprivation therapy; PSMA, prostate-specific membrane antigen; PTV, planning target volume; V95%, volume receiving ≥ 95% of the prescribed dose.; y, years; mo, months; d, days.

The median interval between simulation and first fraction was 16 days (IQR: 10–22; range: 4–87), and median overall treatment duration was 9 days (IQR: 8–10; range: 7–39; see note in text regarding the outlier value). Median PTV1 volume was 63.6 cc (IQR: 47.6–80.1; range: 38.5–127.7 cc), with a median planned V95% coverage of 84.3% (IQR: 80.2–94.9; range: 62.5–98.2%). PTV2 was delivered in 13 patients (41.9%): 25 Gy in 10 cases (76.9%). PTV3 was delivered in 3 patients (9.7%) for macroscopic recurrence (median volume 16.4 cc) and 3 cases (23.1%) corresponding to gross nodal disease. The maximum treatment duration of 39 days reflected one patient who required a planned treatment break due to an intercurrent medical event unrelated to radiotherapy; all remaining patients completed treatment without interruption, with a median duration of 9 days (IQR: 8–10; range excluding outlier: 7–14 days).

### 3.2 PSA kinetics

**Fig. 1:**
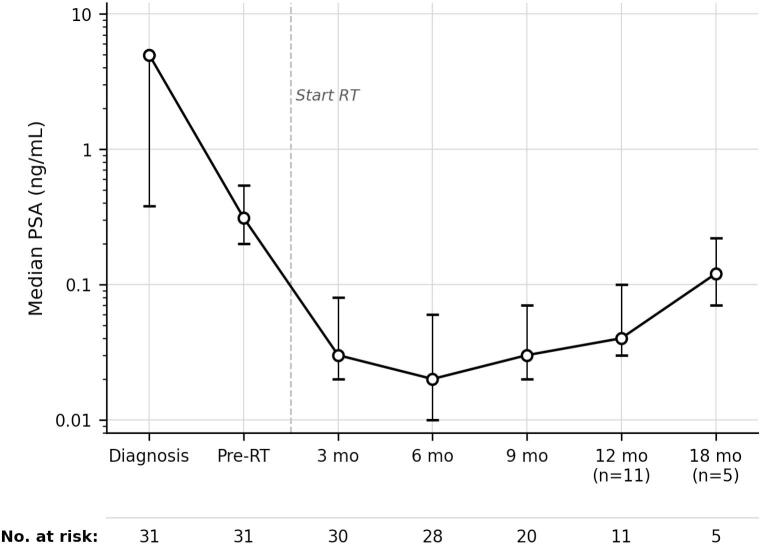
PSA kinetics following MR-guided adaptive SBRT to the prostate bed. Median PSA values (ng/mL) are shown with IQR error bars at each time point on a logarithmic Y-axis. The median PSA value at diagnosis (4.97 ng/mL) is plotted for context. The vertical dashed line indicates the start of radiotherapy. Numbers at risk at each time point are shown below the X-axis: Diagnosis n = 31, Pre-RT n = 31, 3 mo n = 30, 6 mo n = 28, 9 mo n = 20, 12 mo n = 11, 18 mo n = 5. Note: PSA interpretation is significantly confounded by concomitant ADT in 77.4% of patients.

Biochemical response was achieved in all patients. A progressive decline in PSA was observed from treatment initiation (Fig. 1). Median PSA decreased from 0.31 ng/mL pre-RT to 0.03 ng/mL at 3 months, 0.02 ng/mL at 6 months, and 0.03 ng/mL at 9 months. At 12 months (*n* = 11), median PSA was 0.04 ng/mL.

### 3.3 Acute and late toxicity

#### 3.3.1 Genitourinary adverse events

At baseline, 19.4% of patients (6/31) had pre-existing grade 1 GU adverse events, reflecting post-prostatectomy functional status: urinary incontinence G1 in 16.1% (5/31) and urinary frequency G1 in 3.2% (1/31). No patient had grade ≥ 2 GU or any GI adverse events at baseline. At treatment completion (*n* = 30), 33.3% of patients (10/30) experienced any GU adverse event (≥G1). The two most common events were cystitis (30.0%; G1: 26.7%, G2: 3.3%) and urinary incontinence (13.3%; G1: 10.0%, G2: 3.3%). At 3 months (n = 30), urinary incontinence became the predominant finding (26.7%; G1: 16.7%, G2: 10.0%), whereas cystitis decreased to 6.7%. At 6 months (*n* = 23), any-grade GU adverse events were present in 30.4%, primarily attributable to low-grade urinary incontinence (26.1%; G1: 21.7%, G2: 4.3%). Grade ≥ 3 GU adverse events were 0% at all time points. (Table 2).

**Table 2:** Physician-assessed genitourinary adverse events according to CTCAE v5.0, by follow-up time point.

Adverse event domain (CTCAE v5.0)	End of RT (n = 30)		3 Months (n = 30)		6 Months (n = 23)	
	Grade 2	Grade ≥ 3	Grade 2	Grade ≥ 3	Grade 2	Grade ≥ 3
**Genitourinary adverse events**						
Any GU adverse event (≥G2)	3.3% (1/30)	0%	10.0% (3/30)	0%	4.3% (1/23)	0%
Cystitis	3.3% (1/30)	0%	0%	0%	N/A	0%
Urinary incontinence	0%	0%	10.0% (3/30)	0%	4.3% (1/23)	0%
Hematuria	0%	0%	0%	0%	0%	0%
Urinary retention	0%	0%	0%	0%	0%	0%

CTCAE, Common Terminology Criteria for Adverse Events; GU, genitourinary; G2/G3, adverse event grade. *Percentages based on evaluable patients at each time point.

#### 3.3.2 Gastrointestinal adverse events

At treatment completion, only 6.7% of patients developed GI adverse events ≥G1 (G1 diarrhea: 6.7%; G1 rectal tenesmus: 3.3%). At 3 and 6 months, GI adverse events were 0% across all assessed domains. No grade ≥ 3 GI adverse events were documented.

### 3.4 Lower urinary tract symptoms (IPSS)

At baseline, the median total IPSS was 3 points (IQR: 1–7.25; range: 0–18), corresponding predominantly to mild symptoms (77.3%), while 22.7% of patients presented moderate symptoms. The median urinary QoL (item 8) score was 1 (IQR: 0–2).

Pre-treatment urinary continence data were available for 22 patients. At baseline, 22.7% reported leakage less than one in five times, 18.2% less than half the time, and 4.5% required the use of a penile clamp prior to radiotherapy.

**Fig. 2:**
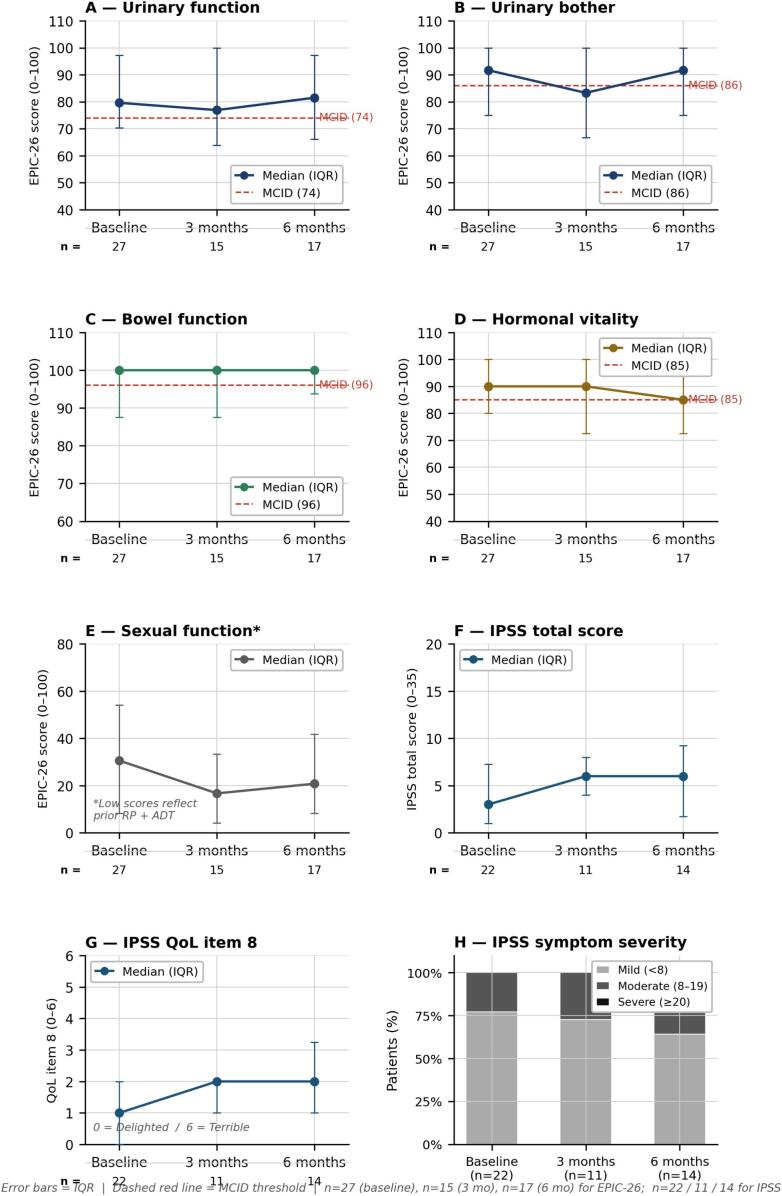
Patient-reported quality-of-life and urinary symptom outcomes following MR-guided adaptive SBRT to the prostate bed. Panels A–E show EPIC-26 domain scores (urinary function, urinary bother, bowel function, hormonal vitality, and sexual function) at baseline, 3 months, and 6 months. Panel F shows IPSS total score (0–35); Panel G shows the IPSS QoL item 8 (0 = delighted; 6 = terrible); Panel H shows the distribution of IPSS symptom severity categories (mild < 8, moderate 8–19, severe ≥ 20) at each time point. Error bars represent IQR. Dashed red lines indicate MCID thresholds where applicable. Panel E: low baseline sexual function scores reflect pre-existing deficits from radical prostatectomy and androgen deprivation therapy. EPIC-26, Expanded Prostate Cancer Index Composite-26; IPSS, International Prostate Symptom Score; MCID, minimal clinically important difference; IQR, interquartile range; RP, radical prostatectomy; ADT, androgen deprivation therapy. (For interpretation of the references to colour in this figure legend, the reader is referred to the web version of this article.)

At 3 months (*n* = 11), median IPSS increased to 6 points (IQR: 4–8; mean: 5.8 ± 2.9), and remained stable at 6 months (*n* = 14), with a median of 6 points (IQR: 1.75–9.25; mean: 6.1 ± 4.8). Overall, no clinically significant deterioration in urinary symptoms was observed, and no patient developed severe LUTS (IPSS ≥20) during follow-up. IPSS outcomes are displayed in Fig. 2(panels F–H).

### 3.5 Patient reported outcomes and QOL

Fig. 2 show changes in EPIC 26. Baseline median urinary domain score was 79.6 points (IQR: 70.4–97.2). At 3 months, scores remained stable (median 76.9; IQR: 63.9–100), and at 6 months increased to 81.5 (IQR: 66.2–97.2). The bowel domain remained consistently at ceiling levels throughout follow-up: median 100 points at all time points. Sexual function scores were low at all time points: median 30.6 at baseline, decreasing to 16.7 in 3 months and 20.8 in 6 months. Hormonal vitality remained stable: median 90 at baseline, 90 at 3 months, and 85 at 6 months. This stability is notable given the high proportion of patients receiving ADT. Urinary bother scores remained high throughout follow-up, with ≥80-point scores in 88.5%, 73.3%, and 82.4% of patients at baseline, 3 months, and 6 months, respectively.

## 4 Discussion

This prospective study reports the clinical experience with MRgRT the prostate bed, providing prospectively collected real-world data on feasibility, acute and subacute adverse events, and QoL outcomes using the validated EPIC-26 instrument within a uniform treatment protocol. The main finding is that MRgRT to the prostate bed delivering 32.5 Gy in 5 fractions over a median of 9 days is feasible and well tolerated, with no grade ≥ 3 adverse events and no clinically meaningful deterioration in QoL domains during early follow-up, although these findings should be interpreted in the context of a limited median follow-up of 8.8 months.

The adverse event outcomes compare favorably with major reference trials in post-prostatectomy radiotherapy. The RADICALS-RT trial established that moderately hypo fractionated radiotherapy yields patient-reported urinary and bowel outcomes comparable to conventional fractionation at 2 years [1], with long-term follow-up confirming these findings while supporting early salvage over adjuvant treatment [2]. Our approach further compresses treatment duration and achieves low early adverse event rates that appear at least comparable to those reported with moderate hypofractionation, although cross-trial comparisons should be made cautiously.

The NRG-GU003 trial demonstrated comparable outcomes with moderate hypofractionation in the postoperative setting, with late grade ≥ 3 adverse events observed in a proportion of patients at long-term follow-up [3]. In contrast, our early results show preserved EPIC-26 urinary scores at 6 months (81.5 vs 79.6 at baseline), although attribution to MR-guided adaptation should be made cautiously given the absence of a control group and the potential confounding effect of concomitant ADT.

From a mechanistic perspective, MR-guided adaptive radiotherapy improves visualization of the relevant anatomical structures, which remain part of the target volume according to contouring guidelines. Nevertheless, it may facilitate treatment re-planning under different bladder filling conditions, which are common in this patient population due to post-surgical residual incontinence and the difficulty of maintaining adequate bladder filling throughout the treatment session. Whether these adaptive replanning advantages translate into clinically meaningful long-term reductions in adverse events remains to be demonstrated in prospective comparative studies.

The SCIMITAR trial provides the most comparable prospective SBRT dataset, reporting late grade 2/3 GU adverse event rates of 31%/4% and GI adverse events of 6.2%/3% at 4 years (a), (b). Similarly, the POPART multicentric prospective study reported salvage SBRT to the prostate bed with a comparable favorable tolerability profile [23]. Importantly, MRgRT delivery was associated with a reduced hazard of GU adverse events compared to CT-SBRT. Our cohort demonstrates a favorable early GU adverse event profile; however, the short follow-up precludes meaningful comparison with late adverse event endpoints reported in SCIMITAR.

Cuccia et al. reported on 22 patients treated with 1.5 T MR-guided adaptive SBRT for prostate or prostate bed re-irradiation, demonstrating technical feasibility and acceptable genitourinary adverse event rates, with grade 2 GU events in 18% of cases [22]. Although not directly comparable to the primary salvage setting due to re-irradiation constraints, it supports the technical feasibility of MR-guided adaptive delivery to this anatomical target.

The SHORTER trial represents the most relevant ongoing randomized benchmark evaluating MRgRT in this setting. While mature results are pending, early reports indicate low rates of severe adverse events [6]. Our findings, particularly the absence of grade ≥ 2 GI adverse events beyond the acute phase, are consistent with these preliminary observations, although definitive conclusions regarding non-inferiority or superiority cannot be drawn.

A key concern in post-prostatectomy SBRT is the potential additive burden of combining pelvic nodal irradiation with ADT. In the SPPORT trial, combined treatment increased acute adverse event rates [5]. In our cohort, despite 77.4% receiving ADT and 41.9% undergoing pelvic nodal irradiation, GI adverse events remained minimal. This observation may reflect the contribution of daily adaptive MR guidance; however, alternative explanations such as patient selection and limited follow-up must also be considered.

Patient-reported outcomes further support the favorable tolerability profile. The modest increase in IPSS scores (median 3 to 6) without severe symptoms is consistent with a transient irritative effect. EPIC-26 urinary and bowel domains remained stable, although interpretation is limited by small evaluable sample sizes at follow-up time points and potential response bias. The preservation of hormonal vitality is notable given the high ADT utilization, but this may be influenced by baseline patient selection and relatively short observation time.

Sexual function outcomes were poor at baseline and declined further, consistent with prior prostatectomy and ADT exposure, and cannot be attributed to radiotherapy effects in this cohort. PSA kinetics demonstrated a rapid decline; however, interpretation of biochemical response is significantly confounded by concomitant ADT and should not be considered a direct surrogate of SBRT efficacy.

A relevant technical observation is the median PTV V95% coverage of 84.3%, which is below conventional planning targets. This likely reflects prioritization of organs of interest constraints during adaptive planning, as was pre-specified in the institutional protocol. While clinically acceptable in this context, this finding raises important questions regarding dose coverage versus toxicity trade-offs that warrant further investigation and explicit reporting in future studies.

Several limitations should be acknowledged. The small sample size limits statistical power and precludes subgroup analyses. The short follow-up does not allow assessment of late toxicity, which may manifest beyond 18–36 months. The absence of a control group limits causal inference regarding the added value of MR guidance. Additionally, treatment heterogeneity, including ADT use and nodal irradiation, introduces confounding factors that complicate interpretation of both toxicity and PSA outcomes.

Despite these limitations, this study provides important early prospective evidence supporting the feasibility and safety of MRgRT in the post-prostatectomy setting and establishes a foundation for future investigations. Future directions include longer follow-up to assess late toxicity and durability of oncologic outcomes, as well as prospective comparative studies against CT-guided SBRT and hypofractionation, ideally incorporating randomized designs to better define the clinical benefit of MR-guided adaptation.

## 5 Conclusions

This study presents prospective evidence on MRgRT to the prostate bed, demonstrating encouraging early safety and tolerability outcomes, with no grade ≥ 3 toxicity and preservation of patient-reported Qol as assessed by EPIC-26. These results provide a basis for further investigation and support the development of multi-institutional prospective studies with longer follow-up and, ideally, comparative designs to better define the clinical benefit of MRgRT.

## CRediT authorship contribution statement

**Daniela Gonsalves Pieretti:** Writing – review & editing, Writing – original draft, Visualization, Methodology, Investigation, Formal analysis, Data curation, Conceptualization. **Fernando López-Campos:** Writing – review & editing, Writing – original draft, Methodology, Investigation, Formal analysis. **Abrahams Ocanto:** Writing – review & editing, Investigation, Formal analysis. **Maria González:** Writing – review & editing, Investigation, Data curation. **Lisselott Torres:** Writing – review & editing, Investigation, Data curation. **Castalia Fernandez:** Writing – review & editing, Investigation, Data curation. **Gloria Sanchez:** Writing – review & editing, Investigation, Data curation. **Gloria Guardia:** Writing – review & editing, Investigation, Data curation. **Alvaro Flores:** Writing – review & editing, Investigation, Data curation. **Jon Andreescu:** Writing – review & editing, Investigation, Data curation. **Laura Viduera:** Validation, Investigation. **Dulce Urias:** Writing – review & editing, Investigation, Data curation. **Ofelia Cabanes:** Writing – review & editing, Investigation, Data curation. **Julia Rogriguez:** Writing – review & editing, Investigation, Data curation. **Sigfredo Romero:** Writing – review & editing, Investigation, Data curation. **Jose Antonio González:** Writing – review & editing, Investigation, Data curation. **Miren Gaztañaga:** Data curation, Investigation and Editing. **Luis Glaria:** Writing – review & editing, Validation, Investigation. **Maia Dzhugashvili:** Writing – review & editing, Investigation. **Stefano Arcangeli:** Writing – review & editing, Supervision, Investigation. **Federica Ferrario:** Writing – review & editing, Supervision, Investigation. **Juan Ignacio Martinez Salamanca:** Writing – review & editing, Resources, Investigation. **Giulio Francolini:** Writing – review & editing, Supervision, Investigation. **Felipe Couñago:** Writing – review & editing, Supervision, Resources, Project administration, Methodology, Investigation, Conceptualization.

## Institutional review board statement

The study was conducted in accordance with the Declaration of Helsinki and approved by the Ethics Committee of Hospital La Princesa (registry #5289). Informed consent was obtained from all participants.

## Funding

This research received no external funding.

## Declaration of competing interest

No author has received employment, consultancy fees, stock ownership, honoraria, paid expert testimony, patent applications, grants, or any other form of funding from any organisation that could be perceived as influencing the work reported in this manuscript. The study received no external funding.

## References

[1] Parker C.C., Clarke N.W., Cook A.D. (2020). Timing of radiotherapy after radical prostatectomy (RADICALS-RT): a randomised, controlled phase 3 trial. Lancet.

[2] Parker C.C., Clarke N.W., Cook A.D. (2024). Timing of radiotherapy (RT) after radical prostatectomy (RP): long-term outcomes in the RADICALS-RT trial (NCT00541047). Ann Oncol.

[3] Pisansky T.M., Hamstra D.A., Ritter M. (2023). Hypofractionated vs. conventionally fractionated radiotherapy for prostate cancer after radical prostatectomy (NRG-GU003): a phase III trial. J Clin Oncol.

[4] Nikitas J., Ballas L.K., Romero T., Lynch C., Ma T.M., Valle L.F. (2025). Patient-reported outcomes with stereotactic intensity modulated radiotherapy after radical prostatectomy: a nonrandomized clinical trial. JAMA Oncol..

[5] Pollack A., Karrison T.G., Balogh A.G. (2022). The addition of androgen deprivation therapy and pelvic lymph node treatment to prostate bed salvage radiotherapy (NRG oncology/RTOG 0534 SPPORT): an international, multicentre, randomised phase 3 trial. Lancet.

[6] Marciscano A.E., Wolfe S., Zhou X.K. (2023). Randomized phase II trial of MRI-guided salvage radiotherapy for prostate cancer in 4 weeks versus 2 weeks (SHORTER). BMC Cancer.

[7] Raison N., Kulkarni M., Elhage O. (2019). Perceptions of patients and healthcare professionals of SBRT for prostate cancer: a qualitative study. BJU Int.

[8] Annamraju H., Yarnold J. (2022). Stereotactic body radiotherapy for prostate cancer: a radiobiological and clinical perspective. Radiol Med.

[9] Kishan A.U., Dang A., Katz A.J. (2019). Long-term outcomes of stereotactic body radiotherapy for low-risk and intermediate-risk prostate cancer. JAMA Netw Open.

[10] Szablewska S., Roszkowski K. (2025). Magnetic resonance-guided adaptive stereotactic radiotherapy in prostate cancer patients: a systematic literature review. Cancers.

[11] Westley R., van Sornsen de Koste J., Iqbal M. (2026). Does overall treatment time impact toxicity and clinical outcomes after MRI-guided stereotactic body radiotherapy to the prostate?. Clin Oncol.

[12] Kishan A.U., Ma T.M., Lamb J.M. (2023). Magnetic resonance imaging–guided vs. computed tomography–guided stereotactic body radiotherapy for prostate cancer: the MIRAGE randomized clinical trial. JAMA Oncol.

[13] Santamaria R., Domínguez A., Martín M. (2024). Image-guided stereotactic body radiotherapy on detectable prostate bed recurrence after prostatectomy in RT-naïve patients. Life.

[14] Yan Y., Li X., Yang X. (2025). Innovative approaches in precision radiation oncology: advanced imaging technologies and challenges which shape the future of radiation therapy. Front Oncol.

[15] Goddard L., Deville C., Hwang W.T. (2025). Real-time adaptive motion management in prostate stereotactic body radiation therapy: clinical and dosimetric analysis of 25 patients. Cureus.

[16] Vargas C.E., Voss M.M., Dodoo C. (2026). Outcomes of a phase II interventional clinical trial of prostate bed stereotactic body radiation therapy for prostate cancer with high-risk features following radical prostatectomy. Int J Radiat Oncol Biol Phys.

[17] González-San Segundo C., Couñago F., Gómez-Iturriaga A. (2019). Androgen deprivation therapy and salvage radiotherapy: are we missing something?. Eur Urol.

[18] Ramey S.J., Yechieli R., Sandler K.A. (2017). Elective nodal irradiation in the salvage treatment of prostate cancer: a systematic review. Int J Radiat Oncol Biol Phys.

[19] Song C., Kim Y.S., Ahn H. (2016). Is there a role for pelvic lymph node irradiation in the salvage setting for patients with biochemical recurrence after radical prostatectomy?. Cancer Res Treat.

[20] Zhang P., Happersett L., Burleson S. (2025). Reduction of postradiation therapy urinary toxicity via intrafractional megavoltage-kilovoltage prostate location monitoring. Int J Radiat Oncol Biol Phys.

[21] Michalski J.M., Lawton C., El Naqa I., Ritter M., O'Meara E., Seider M.J. (2010). Development of RTOG consensus guidelines for the definition of the clinical target volume for postoperative conformal radiation therapy for prostate cancer. Int J Radiat Oncol Biol Phys.

[22] Cuccia F., Rigo M., Figlia V. (2022). 1.5T MR-guided daily adaptive stereotactic body radiotherapy for prostate re-irradiation: a preliminary report of toxicity and clinical outcomes. Front Oncol.

[23] Ferrario F., Franzese C., Faccenda V., Vukcaj S., Belmonte M., Lucchini R. (2023). Toxicity profile and patient-reported outcomes following salvage stereotactic ablative radiation therapy to the prostate bed: the POPART multicentric prospective study. Clin Transl Radiat Oncol.

